# The effect of vibration on the severity of restless legs syndrome in hemodialysis patients

**DOI:** 10.15171/jrip.2017.22

**Published:** 2016-11-21

**Authors:** Habibollah Hosseini, Majid Kazemi, Somayeh Azimpour

**Affiliations:** Rafsenjan University of Medical Sciences, Rafsenjan, Iran

**Keywords:** Vibration, Restless legs syndrome, Hemodialysis

## Abstract

**Introduction:** The restless legs syndrome (RLS) is a neurological disorder in patients undergoing hemodialysis. This syndrome causes individual’s disturbed rest, discomfort, and stress, and secondarily to weakened functioning and disturbance in occupational activities and familial life.

**Objectives:** The present study aimed at investigate the effect of vibration on the severity of the manifestation of symptoms of RLS in hemodialysis patients.

**Patients and Methods:** This is an interventional before-after study conducted on 80 patients with RLS in hemodialysis wards of Yazd hospitals. The samples were selected randomly and intervention was performed on the patients as vibration for 10 minutes three times per week during 4 succeeding weeks. The questionnaire of severity of RLS was completed before the study and at the last day of intervention before and after vibration. The data were analyzed with SPSS 23 using descriptive statistics and paired t test (*P*<0.05).

**Results:** Our findings showed that most patients were at the moderate level of severity of symptoms before (68.8%) and after (78.8%) intervention and there was a significant difference in the mean score of RLS between before (18.99) and after (12.82) intervention (*P*=0.001).

**Conclusion:** Based on the results of this study, it can be concluded that vibration decreases the severity of symptoms of RLS in hemodialysis patients. Hence, it is recommended that vibration be used as a cost-effective and safe procedure to improve the symptoms of RLS in this group of patients.

**Implication for health policy/practice/research/medical education:**

In a study on 80 patients with RLS with the mean age of 61/41 years, we found vibration decreases the severity of symptoms of RLS in hemodialysis patients.

## Introduction

End-stage renal disease (ESRD) is a phase of the disease in which the kidneys are not able to perform the metabolic activities and to maintain the balance of body fluids and electrolytes culminating in a disastrous condition medically termed as uremia. The use of alternative methods as substitutes for the renal functioning is recommended to reduce mortality rate and increase the patients longevity (1) with hemodialysis being the most frequently used substitute (2). In addition to several behavioral changes such as anxiety, depression, seclusion, disease denial, delirium, and hallucination, the consequences of hemodialysis include burning sensation in the body, restless legs syndrome (RLS), feet drop, and even panplegia (3). The RLS is a common sensory-motor disorder (4) manifesting itself as an intensive inclination for moving the feet and sometimes other parts of the body. It may be associated with uneasiness, pain, tingling, and numbness of the feet or other body organs. It is aggravated with rest and inactivity, specifically at night and improves with activity. It may also impair the sleep during night (5). RLS is common in the patients with chronic renal failure. Moreover, the frequency distribution of the RLS is reported to vary from 6.6% to 83% in hemodialysis patients (6). The purpose of treatment of this syndrome is to remove the symptoms, improve life quality, and decrease the untoward sequelae in these patients. The basis of treatment of this syndrome is the use of pharmaceutical and non-pharmaceutical therapies and intensive special care. The pharmaceutical agents used in the treatment of RLS include dopamine agonists (pramipexole, ropinirole, rotigotine), levodopa formation (carbidopa, levodopa), gabapentin, benzodiazepines, opioids, and antiepileptic agents. The recommended non-pharmaceutical factors including regular daily exercise like jogging, stretching movements, hot-water bath, cold-water bath, acupuncture, massaging the affected limps, relaxation exercises (biofeedback or yoga), abstinence from caffeine, alcohol, and tobacco consumption help reduce the symptoms (7). Due to varying success of these pharmaceutical agents and their unexpected after-effects, the provision of reliable non-pharmaceutical alternatives for these patients is rendered as mandatory (4). Vibration causes an increase in the patients’ body temperature and this condition, in turn, leads to increased release of oxygen from myoglobin and hemoglobin, increased muscular blood flow, increased sensitivity of neural receptors, increased nerve velocity, and decreased muscular viscosity. All these factors contribute to improved health, and also the bio-motor and functional indices (8). The case report by Junggi conducted on the effect of whole body vibration on neuropathic pain induced by diabetes in a 71-year-old patient demonstrated that the patient’s neuropathic pain decreased after 8 weeks of application of whole body vibration and the patient’s posture and gait improved significantly (9).

## Objectives

Seeing that no study has directly dealt with the effect of vibration on RLS yet, and most studies have investigated its effect on pain reduction, this study aimed at investigating the effect of vibration on the severity of symptoms of RLS as a non-pharmaceutical method to control this syndrome and its untoward sequelae of unexpected consequences in hemodialysis patients.

## Patients and Methods

This was an interventional before-after study conducted on a population of all the hemodialysis patients in hemodialysis wards of hospitals of Meibod, Ardakan, and also Shahid Rahnemoon hospital in Yazd. A total of 80 patients with RLS were selected by random simple sampling method from among the hemodialysis patients hospitalized in hemodialysis wards. Finally, 4 patients were excluded from the study due to lack of cooperation and the intervention was conducted on just 76 patients. The inclusion criteria were; affliction with chronic renal failure with a history of at least three months of hemodialysis, the presence of RLS, absence of consciousness disturbances, having normal neurological, skeletal, and vascular condition, absence of malignancies, and absence of sores and redness. The exclusion criteria were; patients with catabolic processes like cancer, diabetic neuropathy, consumption of analgesics and opioids, the use of neural and psychological agents, neuromuscular disorders, active or chronic infections, arthritis, and also unwillingness for cooperation to continue treatment. At first, the qualified hemodialysis patients (with inclusion criteria and without exclusion criteria) were screened using the RLS standards. According to this standard, patients with the following four criteria were diagnosed with RLS: frequent movements of feet with feeling of discomfort and lethargy in feet, temporary relief of unpleasant symptoms with moving the feet, onset or aggravation of symptoms with rest or immobility of feet, and onset or exacerbation of symptoms in the evening or at night. Subsequently, the study units were examined by a neurologist to reject the differential diagnoses and it was assured that there were no particular neural or neuropathic disorders and that the study units suffered only from RLS. The goals of the study were completely elucidated for the patients and the patients participated in the study in a fully contended frame of mind. The demographic and clinical information questionnaire included the variables of age, gender, occupation, education level, history of hemodialysis, pain reduction methods, and analgesics consumption. Moreover, the RLS symptoms severity questionnaire consisted of 10 five-scale items each with 0-4 points. The severity of the disorder was classified into five categories on the basis of the obtained points including 0 = without any problems, 1-10 = mild, 11-20 = moderate, 21-30 = severe, and 31-40 = very severe. The validity of the international instrument of RLS was established using the content validity method, and the reliability of the questionnaire has been established to be 97% in the study by Habibzadeh et al (10). The questionnaire was completed by the researcher using the interview method at the beginning of the study. The patients underwent vibration therapy three times per week for one month during their hemodialysis and the vibration was performed at low voltages (Thrive Model 717 A) for 10 minutes on the calves. Next, after 4 weeks, the severity of RLS was measured by questionnaire on the basis of international standards of RLS. Having gleaned the required data, they were imported to SPSS23 and analyzed statistically.

### Ethical issues

The research followed the tenets of the Declaration of Hel­sinki. Informed consent was obtained. This project was approved by the ethical committee of Rafsanjan University of Medical Sciences, Iran (Ir.rums.rec.2015.193) (IRCT ID: IRCT2016022826814N1).

## Results

Our findings revealed that the mean age of the patients under study was 61.41 years. Also, 57.5% of the patients were male, most patients (48.8%) were illiterate, and most of them (43.8%) were unemployed. The findings demonstrated that regarding the severity of symptoms, most patients were at the severe level (31.2%) and the moderate level (68.8%) before the intervention while they were at the mild level (16.2%) and moderate level (78.8%) in this regard after intervention (Table 1).

**Table 1:** Frequency distribution of the severity of RLS before and after interventions

**RLS**	**Before intervention**		**After intervention**	
	**No.**	**%**	**No.**	**%**
Mild	0.0	0.0	13	16.2
Moderate	55	68.8	63	78.8
Severe	25	31.2	0.0	0.0
Subject attrition	0.0	0.0	4	5
Total	80	100	76	100

The findings of the present study showed that the mean and standard deviation (SD) of RLS score were different before intervention by vibration (18.99 ± 3.292) compared to after intervention (12.82 ± 2.726) indicating a significant decrease (*P* = 0.001; Table 2).

**Table 2:** Comparison of RLS mean score before band after vibration

**Parameter**	**No.**	**Mean**	**SD**	***P*** ** value**
RLS before vibration	76	18.99	3.292	0.001
RLS after vibration	76	12.82	2.726	0.001

## Discussion

The present research was an interventional before-after study which aimed at determining the effect of vibration on the severity of symptoms of RLS in hemodialysis patients who presented to the hemodialysis wards in Yazd province hospitals. The findings ultimately proved the positive effects of vibration on reducing the severity of symptoms of RLS. The results of this study showed that 57.5% of the patients under study fell in the 60+ years age group, 32.5% in the 40-60 years age group, and 10% in the 15-40 years age group. The findings also demonstrated that most age groups studied so far fell in the 60+ years age group. The study by Ward et al reported the mean age of the hemodialysis participants as 62.4 years (11). Additionally, Soyoral et al stated the mean age of the hemodialysis patients as 52.28 ± 18.13 years (12). Regarding the point that diabetes and hypertension are the underlying diseases that lead to the emergence of ESRD, and that the incidence of these conditions increases with age (13). Thus, the incidence of chronic renal failure increases with age so that most patients with chronic renal failure belong to the older age groups. This finding is consistent with our results. Moreover, the findings demonstrated that the severity of symptoms in most patients (68.8%) was at the moderate level. In the study by Araujo et al, most patients showed moderate to severe symptoms of RLS (14). Additionally, in the study by Merlino et al, most of the hemodialysis patients had moderate severity of RLS symptoms (15). These findings are consistent with our results. Also, our findings showed that 16.3% of the patients had moderate severity of RLS symptoms after vibration while 78.8% of them suffered from severe symptoms of RLS. These findings indicate an improvement of RLS symptoms after application of vibration. Our findings further revealed that the RLS mean score in the units under investigation was 18.99 before intervention which reduced to 12.82 after intervention by vibration. *T* test also indicated a significant difference in the RLS mean score before and after intervention by vibration (*P* = 0.001). Junggi found that the patients’ pain diminished meaningfully in both feet after 8 weeks of application of vibration. This study showed that vibration therapy reduces the peripheral neuropathic pain and improves the patients’ posture and gait in patients with peripheral neuropathy induced by type II diabetes (9). The review study by Lam et al conducted to investigate the effect of whole body vibration on balance, mobility and falls in older adults concluded that vibration may be effective in improving relatively basic balance ability and mobility among older adults, particularly frailer ones (16). Also, the study by King et al carried out to assess the efficacy of vibration in motor performance of patients with Parkinson disease indicated an improvement in all symptoms, motor control at the time of assessment, and specifically, significant justifiable decrease in rigidity and tremor, and significant increase in gait speed (17). Moreover, in the study by Park et al conducted to survey the effect of whole body vibration on chronic knee osteoarthritis concluded that vibration reduced pain intensity and increased strength of the right quadriceps and dynamic balance performance, and vibration was superior only in pain reduction and similarly effective in strengthening of the quadriceps muscle and balance improvement (18). As mentioned previously, RLS is a kind of side-effect in which the afflicted patient suffers from unpleasant feeling in feet and feels it as tingling, numbness, pain, stretching and jerking, or insect movement on the skin surface so that the patient is obliged to move or stretch their feet to reduce pain. Indeed, vibration leads to increased blood flow in the involved region and ultimately, results in pain reduction in the organ through creating tremor in the organ (19). It can also increase muscular flexibility along with increased maximum muscular power (20). Another potential effect of vibration is its ability for changing the histologic perfusion and balancing the vascular network (21). This leads to increased flow of blood, lymph, and other body fluids which increases the threshold level of edema formation delaying swelling (22).

## Conclusion

On the basis of the findings of this study, it can be concluded that vibration leads to significantly decreased severity of symptoms of RLS in hemodialysis patients. Since RLS is one of the factors contributing to sleep quality disturbances, training patients with measures such as vibration is recommended for controlling and relieving the unpleasant RLS symptoms in hemodialysis patients. Furthermore, regarding the high prevalence of RLS in hemodialysis patients and its effect on life quality, vibration can be used as an adjunct procedure for reducing the RLS symptoms and improving the life and sleep quality of these patients so that they can experience comfort and tranquility in their lives. Finally, future studies should focus on the effect of vibration and massage on fatigue and exhaustion in hemodialysis patients and also their effect on activities of daily living in this community of patients.

## Limitations of the study

Among the limitations of this study were the inability to blind the study and lack of cooperation of some patients.

Low proportion of patients is other limitation of our study.

## Acknowledgments

We hereby appreciate the cooperation and support of the patients and authorities in the hospitals in this study.

## Conflicts of interest

The authors declare no conflict of interest.

## Ethical considerations

Ethical issues (including plagiarism, data fabrication, double publication) have been completely observed by the authors.

## Funding/Support

This article is extracted from MA thesis of nursing Somayeh Azimpour. This study was supported by a grant from Rafsanjan University of Medical Sciences (Thesis #2016).
